# Multi-year monitoring of Piping Plovers (*Charadriusmelodus*) and other shorebirds in The Bahamas

**DOI:** 10.3897/BDJ.11.e96962

**Published:** 2023-01-11

**Authors:** Matthew Jeffery, Walker Golder, Jen Rock, Cheri Gratto-Trevor, Sidney Maddock, Elise Elliott-Smith, Caleb Spiegel, Daniela Linero Triana

**Affiliations:** 1 The Perfect Earth Project, Washington, D.C., United States of America The Perfect Earth Project Washington, D.C. United States of America; 2 North Carolina Coastal Land Trust, Wilmington, United States of America North Carolina Coastal Land Trust Wilmington United States of America; 3 Canadian Wildlife Service, Environment and Climate Change Canada, Sackville, Canada Canadian Wildlife Service, Environment and Climate Change Canada Sackville Canada; 4 Environment and Climate Change Canada, Saskatoon, Canada Environment and Climate Change Canada Saskatoon Canada; 5 ., Buxton, United States of America . Buxton United States of America; 6 United States Geological Survey, Corvallis, United States of America United States Geological Survey Corvallis United States of America; 7 U.S. Fish & Wildlife Service, Hadley, United States of America U.S. Fish & Wildlife Service Hadley United States of America; 8 National Audubon Society, Bogotá, Colombia National Audubon Society Bogotá Colombia

**Keywords:** occurrence, observation, coasts, Piping Plover, shorebirds

## Abstract

**Background:**

The Bahamas provides a wide range of crucial coastal habitats to many declining resident and migratory birds. Amongst these species is the Piping Plover (*Charadriusmelodus*), whose breeding populations are all listed as federally threatened or endangered in the United States and Canada. This species winters in the southern U.S. and the Caribbean, including The Bahamas, spending most of the year on the wintering grounds. Nonetheless, prior to the census data presented here, reports of Piping Plovers from The Bahamas were few and incidental. Therefore, repeated surveys are essential to increase understanding of the distribution, abundance and movement patterns of Piping Plovers and other shorebirds in the Bahamian territory. This dataset provides information on the abundance and distribution of the Piping Plover across multiple islands and much of the suitable habitat that exists in The Bahamas. It also provides some information on the variability of Piping Plover count data and presence of other shorebird species. Furthermore, these data may serve as baseline information on Piping Plover abundance and shorebird site occupancy by which to assess key candidate sites for protection and also future impacts of climate change, such as sea level rise and hurricanes.

**New information:**

The National Audubon Society (NAS), Environment and Climate Change Canada (ECCC) and the United States Geological Survey (USGS) conducted a multi-year shorebird census in The Bahamas. Surveys initiated by ECCC and many other collaborators were also part of a multi-year survival study. Censuses were conducted across 16 different islands between the years 2006 and 2020. These surveys were performed with the cooperation of the Bahamas National Trust (BNT), volunteer biologists and scientists from the United States and Canada. Biologists working with NAS, ECCC and USGS used satellite imagery, historical records and local knowledge from Bahamian residents to identify sites with suitable habitat for Piping Plovers. Experienced researchers visited each site during winter (November-February), identified and counted Piping Plovers and, when possible, other bird species in each of the sampled locations. In total, the resulting database holds 2,684 observations of 62 bird species, of which 77% belong to 24 shorebird species. Approximately 30% of all presence records belong to the Piping Plover. It is important to emphasise that the counts reported in this dataset represent minimum estimates of local shorebird assemblages. Since abundance and distribution of birds vary with changing conditions, representative estimates are best achieved via repeated surveys that reflect a range of conditions including timing (day, year, month), weather (wind direction and speed, precipitation), tide state etc.

## Introduction

Shorebirds are a large avian taxa belonging to the order Charadriiformes, which are commonly called sandpipers, plovers, oystercatchers, avocets, stilts and phalaropes (Brown et al. 2001). They are often considered a group of particular conservation concern due to natural history traits that make them especially vulnerable to threats. These traits include slow reproductive rates, long-distance migration and dependence on a relatively small number of critical migration and wintering sites (Myers et al. 1987, Brown et al. 2001). Such dependance on multiple sites across the hemisphere during their life cycles make them highly sensitive to habitat loss and degradation, disruptions to prey base, hunting, environmental catastrophes (Myers et al. 1987, Brown et al. 2001, Donaldson et al. 2001) and disturbance (Gibson et al. 2018, Palacios et al. 2022). Evidence suggests that long-distance migratory shorebirds have suffered steep declines in the Western Hemisphere since 1970, potentially losing 37-70% of their populations (North American Bird Conservation Initiative 2016, Rosenberg et al. 2019). As threats such as hunting, pollution, habitat loss and degradation and climate change increase, it is essential to identify and protect shorebirds and their habitats throughout their entire range (Myers et al. 1987, Brown et al. 2001, Donaldson et al. 2001).

The Bahamas and Caribbean are widely recognised as important migratory and wintering areas for shorebirds within the Atlantic Flyway, as they may spend half the year or more in these territories (Donaldson et al. 2001, Atlantic Flyway Shorebird Initiative 2015, Cañizares and Reed 2020). Both regions are home to diverse and significant shorebird habitats, including coastal wetlands, mangroves, tidal flats, mudflats, sandy beaches and lagoons. Historical bird surveys in these regions have started to highlight The Bahamas as a potentially critical area for migrating and wintering shorebirds (Cañizares and Reed 2020). In particular, Piping Plovers that breed on the Atlantic coast of North America (*Charadriusmelodusmelodus*) are found by the thousands on beaches, sandy islands, cays and intertidal flats of The Bahamas (Elliott-Smith et al. 2009, Elliott-Smith et al. 2015). Recent research has shown that The Bahamas supports at least 32% of the Atlantic Piping Plover population and up to 19% of the global population of this imperilled species, which is federally listed in the United States and Canada (Species at Risk Act, Statues of Canada 2002, Elliott-Smith et al. 2015, Gratto-Trevor et al. 2016, Wildlife and Fisheries 2021). Therefore, shedding light on shorebird abundance and distribution patterns in The Bahamas is critical to understanding the full life cycle of species that migrate there and to identifying conservation priorities.

The Commonwealth of The Bahamas is an archipelago in the Western Atlantic Ocean, north of the Greater Antilles and southeast of Florida (Buchan 2000). With an area of approximately 13,878 km^2^, The Bahamas consists of more than 700 islands and 2,400 cays (Buchan 2000). The purpose of this paper is to report the results of multiple shorebird surveys, with a particular focus on Piping Plovers, conducted between 2006 and 2020 in The Bahamas. Survey data presented here were collected under three different projects: (1) Shorebird Conservation in The Bahamas, which was carried out by NAS in partnership with BNT; (2) Eastern Canada Piping Plover survival and movement study, initiated by ECCC; (3) International Piping Plover Census, which was coordinated in the Bahamas by the USGS, NAS and BNT.

## General description

### Purpose

This work aimed to improve knowledge about the abundance and distribution of the Piping Plover and other shorebirds in coastal habitats of The Bahamas. This dataset provides valuable information for recognising the diversity of shorebirds present in this country, monitoring changes in species abundance and identifying key conservation sites.

## Sampling methods

### Study extent

The surveys had the primary intention of increasing knowledge about the abundance and distribution of the Piping Plover in The Bahamas. Therefore, experienced researchers conducted sampling in habitats known to support wintering Piping Plovers, such as beaches and sandflats with low density of grasses and other types of vegetation. Sampled sites were located on multiple islands throughout the Bahamas (see geographic coverage description). Surveys were conducted during the wintering period of the Piping Plover in The Bahamas, which can extend from November to the end of February.

### Sampling description

Census sites were selected, based on historical data from areas where Piping Plovers had been documented, analysis of satellite imagery to identify sites with habitat that might support Piping Plovers, local knowledge from Bahamian residents and sites discovered by census teams in the process of completing censuses. Some of these sites were repeatedly censused over the years, while others were progressively added to identify new wintering birds and increase the chance of recording new plover individuals through band resightings. It is important to consider that sites sampled multiple times have the same name, but may have different coordinates due to within-site variation in shorebirds' locations, tide level and habitat changes across the years. At each site, surveys were conducted on foot by researchers skilled in shorebird identification. The censuses conducted under the coordination of ECCC focused only on Piping Plovers, while those coordinated by NAS and the USGS included counts for other bird species when possible, following the same protocol for collecting Plovers' data. Surveyors covered all suitable Piping Plover habitats at each census site, excluding hard-to-reach areas, such as very large tidal flats with no boat access, remote islands and cays and dense mangroves. Additionally, protocols for carrying out surveys were adaptively revised, based on field experiences, and observers were advised to conduct surveys under favourable weather conditions and at medium to high tide levels to increase bird detectability. However, due to the remoteness of many sites, it was not possible to conduct all surveys at ideal times or tide levels and, therefore, counts presented here represent minimum estimates. Finally, in addition to recording birds’ location and abundance, observers also reported the date, time, weather, tidal stage, presence or absence of leg-bands and any colour combinations or alphanumeric leg flags and surveyor information.

### Quality control

Surveys were conducted by experienced shorebird researchers. All records were manually validated, verifying that the information reported in the dataset was consistent with the data collected in the field diaries.

## Geographic coverage

### Description

Censuses were conducted in The Bahamas, primarily in coastal habitats. In particular, the dataset contains occurrence records across 16 different main islands and the associated cays within the archipelago: Abaco, Acklins, Andros, Berry Islands, Bimini, Cat Island, Crooked Island, Eleuthera, Exuma, Grand Bahama, Harbour Island, Inagua, Long Island, New Providence, Ragged Island and San Salvadore.

### Coordinates

20.9167 and 26.9479 Latitude; -79.3011 and -73.2619 Longitude.

## Taxonomic coverage

### Description

The dataset holds occurrence records of 62 bird species, classified in 21 families and 12 orders. The families with the highest number of recorded species were Scolopacidae (16 species), Ardeidae (10 species) and Laridae (9 species). Since most surveys focused on the Piping Plover and the habitat where it occurs, this species has ~ 30% of the total 2,684 presence records. The International Piping Plover Census also focused on other species of plovers. Thus, the number of Piping Plover records are followed by Wilson’s Plover (*Charadriuswilsonia*; ~ 9%) and Black-bellied Plover (*Pluvialissquatarola*; ~ 7%). Species that were encountered during Piping Plover surveys, but in habitat atypical of that used by Piping Plovers (i.e. shrub thickets, birds in flight at the census site, perching birds, raptors etc.) were also recorded. The taxonomic authority used was the American Ornithological Society's Checklist of North American Birds (Chesser et al. 2020).

### Taxa included

**Table:** 

Rank	Scientific Name	Common Name
class	Aves	Birds
species	*Actitismacularius*	Spotted Sandpiper
species	*Ardeaalba*	Great Egret
species	*Ardeaherodias*	Great Blue Heron
species	*Arenariainterpres*	Ruddy Turnstone
species	*Bubulcusibis*	Cattle Egret
species	*Butoridesvirescens*	Green Heron
species	*Calidrisalba*	Sanderling
species	*Calidrisalpina*	Dunlin
species	*Calidriscanutus*	Red Knot
species	*Calidrismauri*	Western Sandpiper
species	*Calidrismelanotos*	Pectoral Sandpiper
species	*Calidrisminutilla*	Least Sandpiper
species	*Calidrispusilla*	Semipalmated Sandpiper
species	*Cathartesaura*	Turkey Vulture
species	*Charadriusmelodus*	Piping Plover
species	*Charadriusnivosus*	Snowy Plover
species	*Charadriussemipalmatus*	Semipalmated Plover
species	*Charadriusvociferus*	Killdeer
species	*Charadriuswilsonia*	Wilson's Plover
species	*Crotophagaani*	Smooth-billed Ani
species	*Egrettacaerulea*	Little Blue Heron
species	*Egrettarufescens*	Reddish Egret
species	*Egrettathula*	Snowy Egret
species	*Egrettatricolor*	Tricolored Heron
species	*Eudocimusalbus*	White Ibis
species	*Falcocolumbarius*	Merlin
species	*Falcoperegrinus*	Peregrine Falcon
species	*Fregatamagnificens*	Magnificent Frigatebird
species	*Geothlypistrichas*	Common Yellowthroat
species	*Haematopuspalliatus*	American Oystercatcher
species	*Himantopusmexicanus*	Black-necked Stilt
species	*Hydroprognecaspia*	Caspian Tern
subspecies	*Larusargentatussmithsonianus*	American Herring Gull
species	*Larusdelawarensis*	Ring-billed Gull
species	*Larusfuscus*	Lesser Black-backed Gull
species	*Limnodromusgriseus*	Short-billed Dowitcher
species	*Limosafedoa*	Marbled Godwit
species	*Megacerylealcyon*	Belted Kingfisher
species	*Mergusserrator*	Red-breasted Merganser
species	*Numeniusphaeopus*	Whimbrel
species	*Nyctanassaviolacea*	Yellow-crowned Night-Heron
species	*Nycticoraxnycticorax*	Black-crowned Night-Heron
subspecies	*Pandionhaliaetuscarolinensis*	Osprey
subspecies	*Pandionhaliaetusridgwayi*	Caribbean Osprey
species	*Pelecanusoccidentalis*	Brown Pelican
species	*Nannopterumauritum*	Double-crested Cormorant
species	*Phalaropustricolor*	Wilson's Phalarope
species	*Phoenicopterusruber*	American Flamingo
species	*Pluvialissquatarola*	Black-bellied Plover
species	*Ralluscrepitans*	Clapper Rail
species	*Rynchopsniger*	Black Skimmer
species	*Setophagadiscolor*	Prairie Warbler
species	*Setophagapalmarum*	Palm Warbler
species	*Setophagapetechia*	Yellow Warbler
species	*Sternadougallii*	Roseate Tern
species	*Sternahirundo*	Common Tern
species	*Tachycinetacyaneoviridis*	Bahama Swallow
species	*Thalasseusmaximus*	Royal Tern
species	*Thalasseussandvicensis*	Sandwich Tern
species	*Tringaflavipes*	Lesser Yellowlegs
species	*Tringamelanoleuca*	Greater Yellowlegs
species	*Tringasemipalmata*	Willet
species	*Vireocrassirostris*	Thick-billed Vireo
kingdom	Animalia	Animals

## Temporal coverage

### Notes

2006-01-09 through 2020-02-08

## Usage licence

### Usage licence

Creative Commons Public Domain Waiver (CC-Zero)

### IP rights notes

This work is licensed under a Creative Commons Attribution Non-Commercial (CC-BY-NC) 4.0 Licence.

Contribution by U.S. Geological Survey authors are work of the United States Government, not subject to copyright protection within the United States, and considered in the public domain in the United States.

## Data resources

### Data package title

Multi-year monitoring of shorebirds in The Bahamas

### Resource link

https://doi.org/10.15468/gwr8mg

### Alternative identifiers

http://ipt.vertnet.org:8080/ipt/resource?r=bhs_shorebirds

### Number of data sets

1

### Data set 1.

#### Data set name

Multi-year monitoring of shorebirds in The Bahamas

#### Data format

Darwin Core

#### Download URL

https://www.gbif.org/dataset/6ce307d0-0456-4b74-b647-db0ace930b26

#### Description

The Bahamas provides a wide range of crucial coastal habitats to many declining resident and migratory birds. Amongst these species is one of the most threatened shorebirds in the United States and Canada, the Piping Plover (*Charadriusmelodus*) (Elliott-Smith et al. 2015). This species winters in the southern US and the Caribbean, including The Bahamas, spending most of the year on the wintering grounds. However, despite various efforts to assess the populations of the Piping Plover and other shorebirds across the Caribbean, their movements, abundance and distribution patterns in this region remain poorly understood (Cañizares and Reed 2020). For this reason, the National Audubon Society, Environment and Climate Change Canada (ECCC) and the United States Geological Survey (USGS) conducted a multi-year shorebird census in The Bahamas. Surveys initiated by ECCC were also part of a multi-year survival study.

Censuses were conducted across 16 different islands between the years 2006 and 2020 (National Audubon Society et al. 2022). These surveys were performed with the cooperation of the Bahamas National Trust, volunteer biologists and scientists from the United States and Canada. Observers counted Piping Plovers and, when possible, other bird species in each of the sampled locations. In total, the dataset holds 2,684 observations of 62 bird species, of which 77% belong to 24 shorebird species. Additionally, 30% of all presence records belong to the Piping Plover, while four species have only one sighting and 29 have ten or fewer records.

It is important to emphasise that the counts reported in this dataset represent minimum estimates of local shorebird assemblages. Since abundance and distribution of birds vary with changing conditions, representative estimates are best achieved via repeated surveys that reflect a range of conditions including timing (day, year, month), weather (wind direction and speed, precipitation), tide state etc.

**Data set 1.** 

Column label	Column description
occurrenceID	Global unique identifier for the occurrence.
modified	Most recent date the data set was modified. Date conforms to ISO 8601-1:2019.
language	Language of the dataset.
license	Statement of the rights assigned to the dataset.
rightsHolder	Organisation that manages data rights.
accessRights	Information about who can access the resource or an indication of use restrictions.
institutionCode	Acronym of the institution having custody of the data or information referred to in the record.
collectionID	An identifier for the dataset from which the record was derived.
bibliographicCitation	Reference indicating how the record should be cited when used.
basisOfRecord	The specific nature of the data record.
eventDate	Date when the occurrence was recorded. Date conforms to ISO 8601-1:2019.
year	The four-digit year in which the occurrence was recorded, according to the Common Era Calendar.
month	The integer month in which the occurrence was recorded.
day	The integer day of the month on which the occurrence was recorded.
eventTime	The time or interval during which an occurrence was recorded. Time conforms to ISO 8601-1:2019.
occurrenceRemarks	Comments or notes about the occurrence.
countryCode	The standard code for the country in which the Location occurs. The code conforms to ISO 3166-1-alpha-2 country codes.
island	The name of the island on or near which the Location occurs.
locality	The specific description of the place.
samplingProtocol	The methods or protocols used during sampling.
samplingEffort	The amount of effort expended during a sampling event.
sampleSizeValue	A numeric value for a measurement of the size of a sample in a sampling event.
sampleSizeUnit	The unit of measurement of the size of a sample in a sampling event.
kingdom	The full scientific name of the kingdom in which the taxon is classified.
order	The full scientific name of the order in which the taxon is classified.
family	The full scientific name of the family in which the taxon is classified.
genus	The full scientific name of the genus in which the taxon is classified.
specificEpithet	The name of the first or species epithet of the scientificName.
infraspecificEpithet	The name of the lowest or terminal infraspecific epithet of the scientificName, excluding any rank designation.
scientificName	The full scientific name, with authorship and date information, if known.
taxonRank	The taxonomic rank of the most specific name in the scientificName.
nameAccordingTo	The reference to the source in which the specific taxon concept circumscription is defined or implied.
scientificNameAuthorship	The authorship information for the scientificName formatted according to the conventions of the applicable nomenclaturalCode.
vernacularName	A common or vernacular name.
individualCount	The number of individuals present at the time of the Occurrence.
occurrenceStatus	A statement about the presence or absence of a Taxon at a Location.
organismRemarks	Comments or notes about the Organism instance.
decimalLongitude	The geographic longitude (in decimal degrees, using the spatial reference system given in geodeticDatum) of the geographic centre of a Location.
decimalLatitude	The geographic latitude (in decimal degrees, using the spatial reference system given in geodeticDatum) of the geographic centre of a Location.
geodeticDatum	The ellipsoid, geodetic datum, or spatial reference system (SRS) upon which the geographic coordinates given in decimalLatitude and decimalLongitude are based.
coordinateUncertaintyInMeters	The horizontal distance (in meters) from the given decimalLatitude and decimalLongitude describing the smallest circle containing the whole of the Location.
dataGeneralizations	Actions taken to make the shared data less specific or complete than in its original form. Alternative data of higher quality may be available on request.
fieldNotes	Text of notes taken in the field.
informationWithheld	Additional information that exists, but that has not been shared in the given record.
